# Geographic Disparities in Readmissions for Peripheral Artery Disease in South Carolina

**DOI:** 10.3390/ijerph19010285

**Published:** 2021-12-28

**Authors:** Brian Witrick, Corey A. Kalbaugh, Lu Shi, Rachel Mayo, Brian Hendricks

**Affiliations:** 1 Department of Public Health Sciences, Clemson University, Clemson, SC 29631, USA; coreyk@clemson.edu (C.A.K.); LUS@clemson.edu (L.S.); rmayo@clemson.edu (R.M.); 2 Department of Bioengineering, Clemson University, Clemson, SC 29631, USA; 3 Department of Epidemiology and Biostatistics, West Virginia University School of Public Health, Morgantown, WV 26505, USA; bmhendricks@hsc.wvu.edu

**Keywords:** peripheral artery disease, disparities, spatial analysis, readmission

## Abstract

Readmissions constitute a major health care burden among peripheral artery disease (PAD) patients. This study aimed to 1) estimate the zip code tabulation area (ZCTA)-level prevalence of readmission among PAD patients and characterize the effect of covariates on readmissions; and (2) identify hotspots of PAD based on estimated prevalence of readmission. Thirty-day readmissions among PAD patients were identified from the South Carolina Revenue and Fiscal Affairs Office All Payers Database (2010–2018). Bayesian spatial hierarchical modeling was conducted to identify areas of high risk, while controlling for confounders. We mapped the estimated readmission rates and identified hotspots using local Getis Ord (G*) statistics. Of the 232,731 individuals admitted to a hospital or outpatient surgery facility with PAD diagnosis, 30,366 (13.1%) experienced an unplanned readmission to a hospital within 30 days. Fitted readmission rates ranged from 35.3 per 1000 patients to 370.7 per 1000 patients and the risk of having a readmission was significantly associated with the percentage of patients who are 65 and older (0.992, 95%CI: 0.985–0.999), have Medicare insurance (1.013, 1.005–1.020), and have hypertension (1.014, 1.005–1.023). Geographic analysis found significant variation in readmission rates across the state and identified priority areas for targeted interventions to reduce readmissions.

## 1. Introduction

Peripheral artery disease (PAD) is a progressive circulatory systems disorder that occurs when there is an obstruction of the peripheral arteries outside of the heart and thoracic aorta that limit the flow of oxygen-rich blood to parts of the body, particularly the lower extremities [1]. PAD can lead to poor quality of life [2] and increased risk of negative outcomes, such as amputation [3,4,5,6,7] or premature death [8,9]. Current estimates indicate that 5% of the global population are affected by PAD, with the prevalence increasing up to 29% in high-risk individuals who are advanced in age and have additional comorbidities [10,11].

Individuals with PAD incur higher health care-related expenditures and have higher rates of hospitalizations than those without PAD [12,13]. Among patients with PAD, people of color, females, older aged adults, and those with comorbid conditions, such as hypertension, diabetes, and heart failure, have higher risk for recurrent hospitalizations or unplanned readmissions [13,14]. Unplanned hospital readmissions are problematic because they are associated with serious medical complications, increased mortality, and high costs [15,16]. However, previous studies have focused mainly on readmissions after revascularization procedures [14], which account for less than half of PAD hospitalizations [17]. Understanding reasons for any unplanned hospital readmissions, including identifying risk factors and geographic areas of increased risk, is essential for improving patient outcomes, decreasing patient costs associated with unnecessary hospital admission and reducing financial penalties imposed on hospital due to high readmission rates [18,19,20,21,22]. 

Spatial analyses allow us to more broadly characterize the extent to which demographic, clinical, and neighborhood level factors impact disparities in risk of readmission in PAD patients and have been implemented in similar studies looking at different populations [22,23,24]. South Carolina is an ideal location to investigate geographic disparities of PAD readmission because of the risk profile of South Carolina residents and a robust repository of all payor healthcare data available at specific geographic levels [25,26]. The aims of this study are to (1) estimate the zip code tabulation area (ZCTA)-level prevalence of readmission among PAD patients in South Carolina and characterize the effect of demographics and comorbidities on ZCTA-level estimates of predicted risk; and (2) identify hotspots of PAD in South Carolina based on estimated prevalence of readmission by ZCTA-level.

## 2. Materials and Methods

### 2.1. Study Population and Data Source

Data for this ecological study were obtained from the South Carolina Revenue and Fiscal Affairs Office (SCRFA) all claims payer database for January 2010 to November 2018. The study population was comprised of all patients with PAD diagnoses, detected using ICD-9/10 codes (Supplementary File S1), aged 18 years or older. Individuals missing zip code of residence were excluded from our analytic sample (*n* = 7, 0.003%). Patient-level data were aggregated to zip code tabulation areas (ZCTAs) by linking spreadsheets with patient zip code of residence to an ESRI Zip code Points shape file and aggregating data to ZCTA level using the 2010 Tiger Line ZCTA shape files [27]. The data were aggregated over time to increase sample size because PAD is relatively uncommon in individuals younger than 50 [28]. Approval for this study was granted by the Clemson University Institutional Review Board (IRB2020-035). 

### 2.2. Study Measurements 

The outcome of this study was 30-day all-cause unplanned readmission prevalence among patients with PAD-related claims. All-cause readmissions were investigated due to the complex, multifaceted impact of PAD and the numerous comorbidities associated with PAD [28]. An unplanned readmission was defined as an inpatient hospitalization within 30 days of discharge from an index event. An index event was admission to an inpatient hospital or an outpatient surgery facility. Following the guidelines of Centers for Medicare and Medicaid services, certain types of care are always considered planned, including transplant surgery, maintenance chemotherapy/immunotherapy, and rehabilitation, and are excluded as an unplanned readmission [29]. Admissions that resulted from transfers between hospitals or units in the hospital were ineligible for inclusion. Additionally, if patients were admitted to the same hospital, on the same day as discharge from index admission, and with the same diagnosis, this was treated as a continuous admission [29]. 

Relevant covariates, identified a priori based on prior knowledge, published literature, and data availability, included demographic and clinical data for patients and were expressed as a proportion at the ZCTA level. Patient characteristics included percent aged 65 and older, percent female, percent non-Hispanic Black, and percent with insurance (Medicare or Medicaid) [13,14,18]. Clinical covariates were identified for patients using ICD-9/10 diagnostic codes (Supplementary File S1). They included rate of diabetes, hyperlipidemia, renal failure, chronic obstructive pulmonary disease (COPD), chronic kidney disease (CKD), hypertension, chronic heart failure (CHF), and coronary artery disease (CAD) [13,14]. Rurality, defined as RUCA score at the ZCTA level, was also included as a covariate due to the higher risk that rural populations have for preventable hospitalizations [30,31,32]. Rurality was defined using Rural-Urban Community Area Codes, where a ZCTA was categorized as urban if the RUCA code was 1.0, 1.1, 2.0, 2.1, 3.0, 4.1, 5.1, 7.1, 8.1, and 10.1 and rural if it was any other value [33]. 

### 2.3. Statistical Analysis 

Descriptive statistics were reported for all demographic characteristics and comorbid conditions by readmission status. Normally distributed continuous variables were reported as mean ± standard deviation and statistical differences were identified using t-test. Categorical variables were presented as proportions and the chi-square test was performed to identify differences by readmission status. Analyses were conducted using SAS version 9.4 (SAS Institute Inc., Cary, NC, USA) and statistical significance was determined at a *p* level of 0.05. 

Hierarchical Bayesian spatial models were conducted to identify associations between 30-day unplanned readmission and covariates using the INLA package in R version 3.4.2 [34,35,36]. To adjust for correlated and uncorrelated spatial heterogeneity and generate stable and reliable prevalence rates, we applied the conditional autoregressive Besag-York-Mollie (BYM) model [37]. More information about this model, including mathematical specifications, can be found elsewhere [38,39,40]. In short, this model includes a spatial random effect, which accounts for ZCTA level spatial dependence (e.g., clustering) of readmission prevalence and a non-spatial random effect, which accounts for any residual ZCTA level variation that is not spatially structured. The BYM is a Poisson model where the number of individuals with a readmission is the dependent variable, the total number of total patients with PAD related claims over the study period at the ZCTA level is the offset variable, and relevant covariates are adjusted for. The BYM model then weights the prevalence rate of specific ZCTA towards the prevalence of a neighboring ZCTA. The neighborhood structure was defined as ZCTA sharing a common edge or border [38]. Following what has been done in recently published studies utilizing spatial BYM models to examine risk of health events and inform public health practice, our priors for the models were fixed (mean = 0, precision = 0.001) [41]. Parameter estimates from modeling were transformed through exponentiation to interpret effects as prevalence rate ratios, as has been done in previous spatial epidemiological modeling studies [42], and influential covariates were assessed using 95% credible intervals (Crl), where intervals crossing one indicate the corresponding variable is non-influential. 

To pinpoint areas for future public health interventions, spatial trends in fitted prevalence of 30-day unplanned readmissions were identified using local Getis Ord (G *) in R using the spdep package and default queen contiguity spatial weight [39,43]. Hotspots were regarded as ZCTAs with Z-scores in the highest mapped class. Alternatively, cold spots were ZCTAs with Z-scores in the lowest mapped class. 

## 3. Results

From January 2010 to November 2018, there were 232,731 individuals admitted to an inpatient hospital or outpatient surgery facility in South Carolina with PAD. Of these, 30,366 (13.1%) experienced an unplanned readmission to an inpatient hospital within 30 days of discharge (Table 1). Overall, PAD patients were more likely to be males who resided in an urban area and had an average age of 65.7 years (SD 14.2). Patients who experienced a 30-day unplanned readmission were more likely to be non-Hispanic Black (37.0% vs. 27.6%) and have Medicare (73.5% vs. 62.1%) or Medicaid (7.5% vs. 6.0%) insurance. Furthermore, patients with a 30-day unplanned readmission were more likely to have comorbidities, such as diabetes (72.5% vs. 53.1%), COPD (47.2% vs. 27.0%), and hyperlipidemia (77.2% vs. 60.0%). 

**Figure 1:**
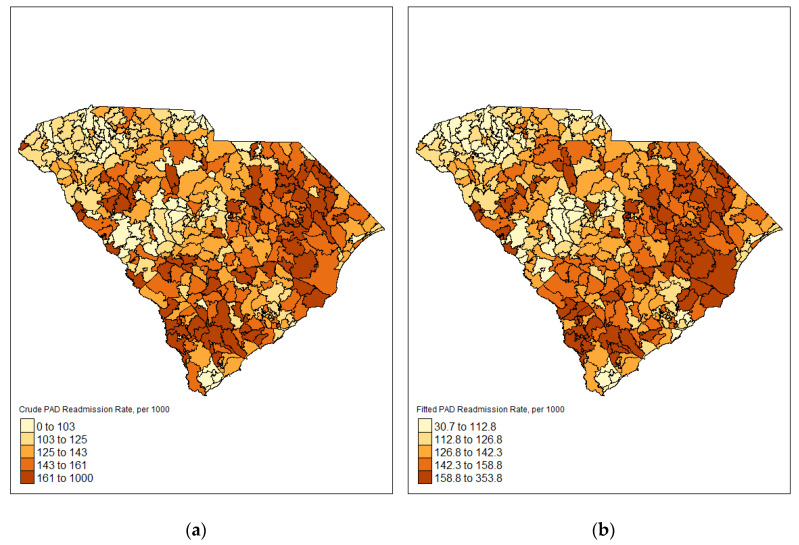
ZCTA level choropleth maps displaying crude and fitted readmission prevalence (**a**) Crude prevalence of PAD readmissions (**b**) Fitted prevalence of PAD readmissions.

**Figure 2:**
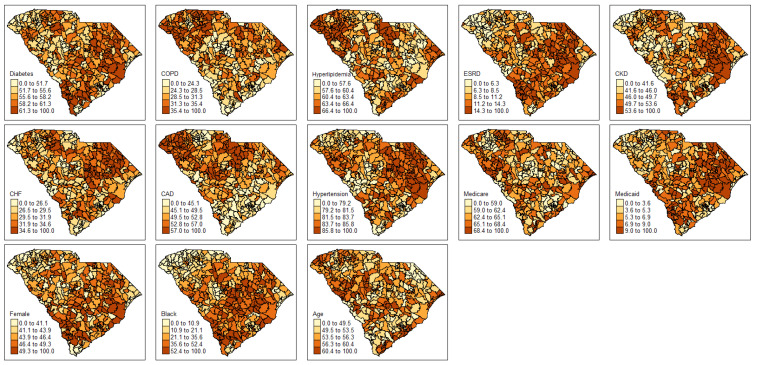
Choropleth maps of percent of comorbid conditions at the ZCTA level.

The crude prevalence of readmission among PAD patients varied geographically throughout the state of South Carolina (Figure 1). Readmission rates were lowest in the northwest region of the state and highest in the south and eastern parts of the state. After fitting the readmission models for demographic characteristics and comorbidities, estimates of readmission prevalence ranged from 35.2 per 1000 to 370.7 per 1000 (Figure 1). Fitted readmission rates remained lowest in the northwest region of the state and highest in the south and eastern part of the state. Covariates were mapped by percent of PAD patients for comorbidities and demographic characteristics (Figure 2). Each map was given its own legend to account for wide variability of mapping classification for each covariate. Some covariates, such as age 65 and older and public insurance varied throughout the state with ZCTAs of high and low percentages. Other covariates, such as diabetes, renal kidney failure, CKD, CHF, hypertension, African American and female followed similar patterns to the fitted readmission rates with higher percentages in the southeast part of the state and lower percentages in the northwestern part of the state. Conversely, COPD, hyperlipidemia, and CAD had higher percentages in the northwest part of the state and lower percentages in the southern parts of the state. 

The risk of readmission among PAD patients is higher in ZCTAs with a greater percentage of patients on Medicare insurance and those with hypertension (Table 2). Conversely, the risk of readmission is lower in ZCTAs with a great percentage of adults aged 65 and older. The percentage of patients who are non-Hispanic Black is marginally significant, with higher risk of readmission among ZCTAs with a greater percentage of non-Hispanic Blacks. 

**Figure 3:**
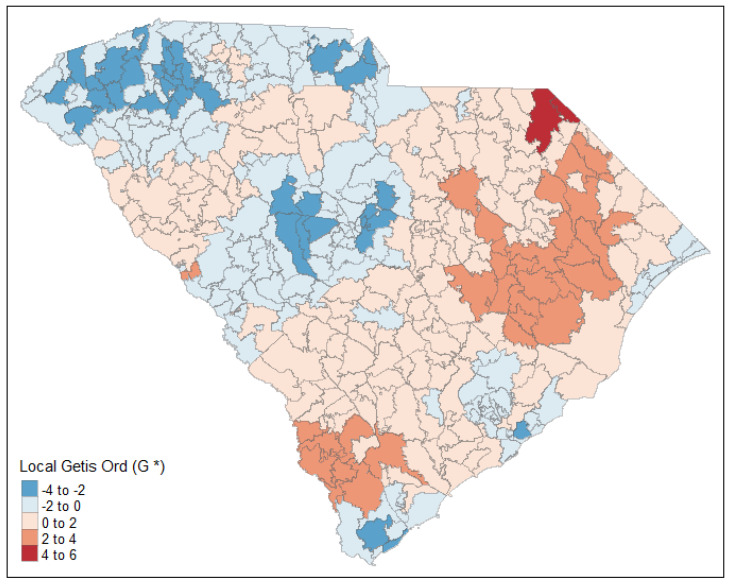
Hot spot of estimated readmission prevalence.

After factoring in the effects of demographic characteristics and comorbidities, there is significantly clustering in readmission rates throughout the state of South Carolina (Figure 3). ZCTA clusters of lower readmission rates, indicated by lighter shades of blue, are in the northwestern and central areas of the state. Alternatively, significant clustering of ZCTAs with higher readmission rates, as indicated by deeper shades of red, are in the southern and eastern areas of the state. In particular, there was a significant hot spot of higher readmission rates near the North Carolina border in Marlboro County.

## 4. Discussion

The aims of this study were to estimate the zip code tabulation area (ZCTA)-level prevalence of readmission among PAD patients in South Carolina, understand the role of demographics and comorbidities on predicted risk, and identify hotspots of PAD based on estimated prevalence of readmission by ZCTA-level. Overall, we found adjusted readmission rates for PAD patients ranged from 35.3 per 1000 to 370.7 per 1000 PAD patients and the risk of having a readmission was significantly associated with the percentage of PAD patients who are younger, have Medicare insurance, and have hypertension. Furthermore, our spatial model identified significant geographic variation in readmission rates throughout the state of South Carolina that persisted after adjusting for important readmission risk factors.

The study of geographic variation of hospital readmissions is important to understanding gaps in quality and capacity of care for patients and our study represents a significant contribution to a small literature [43]. A recent systematic review found geographic variation for ambulatory care-sensitive conditions in 90% of studies reviewed [21]. Studies in France and Canada found considerable geographic variation in hospital readmissions after adjusting for demographic covariates and spatial effects [22,24]. Our study is among the first in the United States to model the prevalence of 30-day hospital readmissions among PAD patients across a state by addressing demographics, medical covariates, and spatial effects [14,25,26]. The high readmission rates in our study (13.1%) highlight the need for further research on preventing additional hospitalizations in areas similar to South Carolina with reduced access to high quality care [25,44]. 

We identified clusters of ZCTAs with high readmission rates in the southern and eastern area of South Carolina. ZCTAs with high readmission rates could be prioritized for interventions to prevent or reduce readmissions among patients with PAD. The results from our study can be used to implement cost-effective interventions designed for location-specific risk groups in these “hot spots”. Community-based multidisciplinary transitional care programs are effective at reducing hospital readmissions in targeted populations that are similar to patients with PAD, including older adults and those with multiple comorbid conditions, and may reduce subsequent hospitalizations in patients with PAD [45,46]. 

Our study found that hypertension, which is a significant risk factor in the development and progression of PAD [28,47], was also associated with increased rehospitalizations. Blood pressure management in PAD patients is often described as a balance between risk factor modification and limb perfusion and is important to prevent poor outcomes and yet there is much debate about the best approach to treating hypertension in PAD patients [48]. Although the American College of Cardiology/American Heart Association hypertension guidelines have recommended a treatment target of <130/80 mm Hg, a post hoc analysis of the ALLHAT trial (Antihypertensive and Lipid-Lowering Treatment to Prevent Heart Attack Trial) found a J-shaped relationship between systolic blood pressure and PAD events, with both low and high blood pressure being associated with a higher risk of PAD events [48,49,50]. These studies highlight the difficulty of managing hypertension in this population [46]. There is a need for further investigation into the ideal blood pressure range for patient with PAD, not only to address PAD, but also to better understand other adverse events that lead to hospital readmissions [51]. 

Racial disparities associated with PAD are well-documented and non-Hispanic black patients are three times as likely to have PAD and have 36% higher odds of being readmitted to the hospital after a revascularization intervention [14,52]. We found an increased association between race and all unplanned hospital readmissions, further emphasizing the racial disparity in PAD related care [53,54]. In South Carolina, higher readmission rates among communities of color are problematic in a state where the population of non-Hispanic Black individuals is nearly twice the national census (27% vs. 14%) [55]. Healthcare practitioners in South Carolina should be particularly cognizant of the racial disparities in PAD-related care and work to improve care for communities of color. 

Our study found that Medicare insurance was associated with increased hospital readmissions in patients with PAD. The Hospital Readmissions Reduction Program has imposed considerable Medicare payment penalties on hospitals with higher-than-expected readmission rates [56]. In states like South Carolina with high proportions of residents on Medicare (SC: 21%) [57] that also have high prevalence of PAD, the high readmission rates observed in our population could translate to serious strain on a state healthcare system. We recommend that hospitals streamline care coordination across the continuum to ensure that patients are receiving appropriate care in the best setting. Hospitals should utilize new care strategies, such as telehealth, to provide post-acute services to patients and reduce readmissions and minimize financial penalties [58]. 

While it may seem counterintuitive that young age is associated with an increased risk of hospital readmission, the prevalence of premature PAD, characterized by disease diagnosis before the age of 50, has been gradually increasing over recent years [59]. Patients with premature PAD have more severe symptoms, a higher prevalence of comorbidities, and a greater risk of disability or death due to a more aggressive disease course [60]. Managing patients with premature PAD involves a multidisciplinary approach with collaboration from numerous healthcare practitioners and a focus on lifestyle interventions and medical management [59]. Primary care and hospital-based healthcare practitioners need to ensure that these patients are receiving appropriate disease management to reduce the risk of unnecessary hospital admissions.

This study has several strengths. First, the use of hierarchical Bayesian spatial modeling allows us to obtain stable and accurate readmission prevalence estimates for all ZCTAs, while controlling for medical conditions and demographic characteristics. Bayesian spatial modeling is widely used in disease mapping due to its ability to easily handle structured data and give more accurate estimates by including information from adjacent regions [61,62]. Additionally, spatial mapping illustrates precise prevalence estimates by ZCTAs and identifies statistically significant clusters of high and low readmission prevalence, which allows for a better understanding of the spatial pattern and distribution of readmissions in South Carolina. Finally, this study was population-based and includes all payer data throughout the entire state. The comprehensive dataset spans multiple years and allows for the ability to continuously obtain data on patients in South Carolina, even if they move within the state, change insurance status, or change employment. This allows for generalizations across the state, which will aid in policy decision making. 

This study has several limitations that should be noted. First, this study is based on administrative hospital discharge data, which can have potential bias because of variation in coding practices between hospitals and medical practices. Additionally, only diagnosed medical conditions are included in the study, so patients may have undiagnosed medical conditions that are not controlled for. Nevertheless, this approach is consistent with other studies using administrative data. Second, there is the potential for a geographic edge effect, which is caused by patients who reside in ZCTAs near the state border seeking care in out-of-state hospitals. This is common in South Carolina due to nearby hospitals in Georgia and North Carolina. This may lead to an underestimation of predicted risk in ZCTAs near the border. 

## 5. Conclusions

This study identified geographic variation of the prevalence of 30-day hospital readmission among PAD patients in South Carolina, even after adjusting for demographic and medical covariates. This is especially important given the high-risk of PAD in South Carolina and the strain that hospital readmissions place on the healthcare system. Although more research is recommended to understand why these variations exists, targeted interventions should be implemented in identified priority areas.

## Figures and Tables

**Table 1:** Patient-level characteristics of all-cause hospital 30-day readmission.

	All Patients (232,731)	No 30-Day Readmission (202,365)	Any 30-Day Readmission (30,366)	*p* Value
Age, mean (SD)	65.74 (14.23)	65.56 (14.36)	66.93 (13.31)	<0.001
Gender (%)				<0.001
Male	127,150 (54.63)	109,813 (54.26)	17,337 (57.09)	
Female	105,581 (45.37)	92,552 (45.74)	13,029 (42.91)	
Race/Ethnicity (%)				<0.001
Non-Hispanic White	159,372 (68.48)	140,743 (69.55)	18,629 (61.35)	
Non-Hispanic Black	67,104 (28.83)	55,872 (27.61)	11,232 (36.99)	
Hispanic	1421 (0.61)	1291 (0.64)	130 (0.43)	
Other	4834 (2.08)	4459 (2.20)	375 (1.23)	
Insurance (%)				<0.001
Private	47,610 (20.46)	43,788 (21.64)	3822 (12.59)	
Medicare	148,003 (63.59)	125,672 (62.10)	22,334 (73.54)	
Medicaid	14,474 (6.22)	121,187 (6.02)	2287 (7.53)	
No Insurance	12,351 (5.31)	11,324 (5.60)	1027 (3.38)	
Other	10,292 (4.42)	9393 (4.64)	899 (2.96)	
Rurality (RUCA categorization C)				<0.001
Rural	70,626 (30.35)	60,512 (29.90)	10,114 (33.31)	
Urban	162,105 (69.65)	141,853 (70.10)	20,252 (66.69)	
Diabetes	129,417 (55.61)	107,406 (53.08)	22,011 (72.49)	<0.001
COPD	69,022 (29.66)	54,681 (27.02)	14,341 (47.23)	<0.001
Hyperlipidemia	144,850 (62.24)	121,397 (59.99)	23,453 (77.23)	<0.001
Renal Failure	23,361 (10.04)	17,191 (8.50)	6170 (20.32)	<0.001
Chronic Kidney Disease	110,195 (47.35)	89,234 (44.10)	20,961 (69.03)	<0.001
CHF	70,572 (30.32)	53,687 (26.53)	16,885 (55.60)	<0.001
CAD	119,615 (51.40)	99,401 (49.12)	20,214 (66.57)	<0.001
Hypertension	191,242 (82.17)	162,263 (80.18)	28,979 (95.43)	<0.001

COPD = chronic obstructive pulmonary disease; CHF = chronic heart failure; CAD = coronary artery disease.

**Table 2:** ZCTA Level prevalence rate ratios for covariates.

ZCTA Variables	Prevalence Rate Ratio (95% CI)
Rural	1.032 (0.980–1.086)
Percent CAD	0.997 (0.993–1.002)
Percent Chronic Kidney Disease	1.004 (0.998–1.011)
Percent Diabetes	1.001 (0.993–1.008)
Percent Medicare	1.013 (1.005–1.020) *
Percent Medicaid	1.008 (0.998–1.019)
Percent Hyperlipidemia	1.001 (0.995–1.007)
Percent 65 and older	0.992 (0.985–0.999) *
Percent Black	1.001 (1.000–1.004)
Percent CHF	1.004 (0.997–1.012)
Percent COPD	1.003 (0.997–1.012)
Percent Female	1.003 (0.997–1.010)
Percent Renal Failure	1.001 (0.994–1.014)
Percent Hypertension	1.014 (1.005–1.023) *

COPD = chronic obstructive pulmonary disease; CHF = chronic heart failure; CAD = coronary artery disease * Indicates substantially influential credible intervals for model covariates.

## Data Availability

Restrictions apply to the availability of these data. Data was obtained from the South Carolina Revenue and Fiscal Affairs Office and are available from the authors with the permission of the South Carolina Revenue and Fiscal Affairs Office.

## Supplementary Materials

The following supporting information can be downloaded at: https://www.mdpi.com/article/10.3390/ijerph19010285/s1, File S1: International Classification of Diseases (ICD) 9 and 10 codes.
