# Supplementary material for: Geographic Disparities in Readmissions for Peripheral Artery Disease in South Carolina
Source: Int J Environ Res Public Health. 2021 Dec 28;19(1):285. doi: 10.3390/ijerph19010285 (PMC8751080; doi:10.3390/ijerph19010285)
Supplement: Supplementary file 1 [file ijerph-19-00285-s001.zip › ijerph-1470061-supplementary.pdf]

International Classification of Diseases (ICD) 9 and 10 codes

Diabetes

ICD-9 codes:

249, 250, 250.0, 250.1, 250.2, 250.3, 250.4, 250.5, 250.6, 250.7, 250.8, 250.9

ICD-10 codes:

E100, E101, E106, E108, E109, E110, E111, E116, E118, E119, E120, E121, E126, E128, E129, E130, E131, E136, E138, E139, E140, E141, E146, E148, E149, E102, E103, E104, E105, E107, E112, E113, E114, E115, E117, E122, E123, E124, E125, E127, E132, E133, E134, E135, E137, E142, E143, E144, E145, E147

Chronic Obstructive Pulmonary Disease

ICD-9 codes:

490, 491.0, 491.1, 491.2, 491.21, 491.22, 491.8, 491.9, 492, 492.8, 494, 494.0, 494.1, 496, 493.00, 493.01, 493.02, 493.10, 493.11, 493.12, 493.20, 493.21, 493.22, 493.81, 493.82, 494.90, 493.90, 493.92

ICD-10 codes:

J40, J41, J42, J43, J44, J45, J47

Hypercholesterolemia ICD-9 codes:

272

ICD-10 codes:

E78, E881, E7521, E7522, E75249, E770, E771, E8889

Renal failure

ICD-9 codes:

403.01, 403.11, 403.91, 404.02, 404.03,
404.12, 404.13, 404.92, 404.93, 585.5, 585.6, 586, 593.81,V45.11, V56

ICD-10 codes:

I120, I1311, I132, N185, N186, N19, N280, Z992, Z4931, Z4901, Z4902, Z4932

Chronic kidney disease

ICD-9 codes:

249.4, 250.4, 403, 403.00, 403.10,403.90, 404, 404.00, 404.01, 404.10, 404.11, 404.90, 404.91, 581, 581.8, 582, 583, 585.1-585.4, 585.9

ICD-10 codes

E0821, E0921, E0865, E1122, E1129, E1029, E1022, E1121, E1165, E1021, E1065, I129, I120, I130, I1311, I132, I1310, N044, N022, N043, N040, N08, N048, N049, N032, N033, N034, N038, N08, N039, N059, N052, N055, N171, N172, N058, I129, I1310, I130, N181, N182, N183, N184, N189

Congestive heart failure

ICD-9 codes:

398.91, 402.01, 402.11, 402.91, 404.01, 404.03 404.11, 404.91, 404.13, 404.93, 425.4, 425.5, 425.7, 425.8, 425.9, 428

ICD-10 codes

I43,I50,I099,I110,I130,I132,I255,I420,I425,I426, I427,I428,I429,P290

Coronary artery disease

ICD-9 codes:

410, 411, 412, 414, 414.0, 429.0,429.1, 429.2, 429.3, 429.4, 429.5, 429.6, 429.7, 429.71,

429.79, 429.8, 429.81, 429.82, 429.89, 429.9, v45.81, V45.82

ICD-10 codes:

I2109, I2119, I2111, I2129, I214, I213, I219, I21A1, I21A9, I241, I200, I240, I248, I252, I2510, I25810, I25811, I25812, I253, I2541, I2542, I2582, I2584, I255, I2589, I259, I514, I515, I517, I970, I97110, I97130, I97190, I511, I512, I510, I230, I5189, I513, I519, Z951, Z955, Z9861

Hypertension

ICD-9 codes:

401,402,403,404,405

ICD-10 codes

I10, I11, I12, I13, I14, I15, I16

Peripheral Artery Disease

ICD-9 codes:

440.22, 440.23, 440.24, 707.10, 707.11, 707.12, 707.13, 707.14, 707.15, 707.16, 707.17, 707.18, 707.19, 785.4, 440.2, 440.20, 440.21, 440.22, 440.23, 440.24, 440.29, 440.3, 440.0, 440.30, 440.31, 440.32, 440.9, 249.7, 249.71, 250.7, 250.71, 250.72, 250.73, 443.1, 443.81, 443.9, 444.22, 444.81, 785.4,

ICD-10 Codes:

I70.229, I70.25, I70.269, L97.909, L97.109, L97.209, L97.309, L97.409, L97.509, L97.809, I96, I70.209, I70.219, I70.229, I70.25, I70.269, I70.299, I70.0, I70.399, I70.499, I70.599, I70.90, I70.91, E08.51, E09.51, E13.59, E08.51, E08.65, E09.51, 11.51, E10.51, E11.51, E11.65, E10.51, E10.65, I73.1, I79.8, I73.9, I74.5, I74.3, I96,
